# Six *Medicago truncatula* Dicer-like protein genes are expressed in plant cells and upregulated in nodules

**DOI:** 10.1007/s00299-016-1936-8

**Published:** 2016-01-29

**Authors:** Aleksander Tworak, Anna Urbanowicz, Jan Podkowinski, Anna Kurzynska-Kokorniak, Natalia Koralewska, Marek Figlerowicz

**Affiliations:** Institute of Bioorganic Chemistry, Polish Academy of Sciences, Noskowskiego 12/14, 61-704 Poznan, Poland; Institute of Computing Science, Poznan University of Technology, Piotrowo 2, 60-965 Poznan, Poland

**Keywords:** Dicer-like (DCL), Legume, *Medicago truncatula*, MicroRNA (miRNA), Nodule, Small interfering RNA (siRNA)

## Abstract

*****Key message***:**

**Here we report the existence of six putative Dicer-likegenes in the*Medicago truncatula*genome. They are ubiquitously expressed throughout the plant and significantly induced in root nodules.**

**Abstract:**

Over the past decade, small noncoding RNAs (sncRNA) have emerged as widespread and important regulatory molecules influencing both the structure and expression of plant genomes. One of the key factors involved in sncRNA biogenesis in plants is a group of RNase III-type nucleases known as Dicer-like (DCL) proteins. Based on functional analysis of DCL proteins identified in *Arabidopsis thaliana*, four types of DCLs were distinguished (DCL1-4). DCL1 mainly produces 21 nt miRNAs. The products generated by DCL2, DCL3, and DCL4 belong to various classes of siRNAs that are 22, 24 and 21 nt in length, respectively. *M. truncatula* is a model legume plant closely related to many economically important cultivable species. By screening the recent *M. truncatula* genome assembly, we were able to identify three new DCL genes in addition to the MtDCL1-3 genes that had been earlier characterized. The newly found genes include *MtDCL4* and two *MtDCL2* homologs. We showed that all six *M. truncatula* DCL genes are expressed in plant cells. The first of the identified *MtDCL2* paralogs encodes a truncated version of the DCL2 protein, while the second undergoes substantial and specific upregulation in the root nodules. Additionally, we identified an alternative splicing variant of MtDCL1 mRNA, similar to the one found in *Arabidopsis*. Our results indicate that DCL genes are differently activated during *Medicago* symbiosis with nitrogen fixing bacteria and upon pathogen infection. In addition, we hypothesize that the alternative splicing variant of MtDCL1 mRNA may be involved in tissue-specific regulation of the DCL1 level.

**Electronic supplementary material:**

The online version of this article (doi:10.1007/s00299-016-1936-8) contains supplementary material, which is available to authorized users.

## Introduction

Small noncoding RNAs (sncRNAs) including microRNAs (miRNAs) and several types of small interfering RNAs (siRNAs) are among the most important factors influencing both the structure and expression of eukaryotic genomes (Szweykowska-Kulińska et al. 2003). SncRNAs are involved in the regulation of numerous biological processes, such as development, stress response and pathogen defense (Kurzyńska-Kokorniak et al. 2009; Jackowiak et al. 2011; Castel and Martienssen 2013; Martínez de Alba et al. 2013). Biogenesis of both miRNAs and siRNAs depends on a group of endonucleolytic enzymes that are members of the RNase III family, known as Dicer proteins in animals (Kurzynska-Kokorniak et al. 2015) and Dicer-like (DCL) proteins in plants (Bologna and Voinnet 2014). *Arabidopsis thaliana,* a model dicot plant, encodes four DCL proteins (AtDCL1-4). Each of these represents a distinct type of DCL found in plants. All four types are believed to have diverged early in plant evolution (Mukherjee et al. 2013). AtDCL1 is mainly responsible for the production of 21 nt miRNAs, while AtDCL2, AtDCL3, and AtDCL4 produce different classes of siRNAs (Xie et al. 2004). However, the functions of all DCL enzymes are partially redundant (Gasciolli et al. 2005). In many plants, duplications of the genes encoding the DCL proteins have been observed. For example, the rice genome encodes two DCL2 and two DCL3 isoforms (Kapoor et al. 2008), whereas in soybean, two isoforms each of DCL1, DCL2, and DCL4 were identified (Curtin et al. 2012).

Plant DCL proteins share a common domain architecture with their animal counterparts. A typical DCL protein contains an N-terminal helicase domain (DExD/H-box and helicase-C subdomains) followed by DUF283 (domain of unknown function, known also as Dicer dimerization domain), PAZ (Piwi-Argonaute-Zwille), tandem RNase III domains and one or two C-terminal dsRBDs (dsRNA binding domains). The major catalytic activity of DCL enzymes is provided by the two RNase III domains, which cleave dsRNA substrates and release short RNA duplexes with 2 nt 3′ overhangs and phosphorylated 5′ ends. In the miRNA biogenesis pathway, DCL1 produces duplexes formed by two 21 nt RNA strands: miRNA and a complementary passenger strand referred to as miRNA*. Both PAZ and the helicase domains are known for their role in proper positioning of the sncRNA precursor within the enzyme (Macrae et al. 2006; Gu et al. 2012), while the helicase also allows processive cleavage of longer substrates (Cenik et al. 2011; Welker et al. 2011).

The expression patterns of different DCL genes vary depending on the tissue or environmental conditions (Liu et al. 2009; Capitão et al. 2011). Two miRNAs (miR162 and miR838) were shown to negatively regulate the level of *DCL1* expression in *Arabidopsis*. Both are involved in negative feedback loops that control the level of DCL1 in plant cells. Conserved miR162 targets the AtDCL1 mRNA within the exon 12–13 junction. The biogenesis of non-conserved miR838 located in intron 14 of the AtDCL1 mRNA precursor was proposed to interfere with proper splicing of AtDCL1 transcript (Xie et al. 2003; Rajagopalan et al. 2006). However, the latter phenomenon was discovered in a single plant species and the question of whether this is a general mechanism or specific to the selected species has not yet been addressed.

*Medicago truncatula* is a model legume plant closely related to many economically important cultivable species. A unique feature of legumes is their ability to fix nitrogen from the atmosphere, which is facilitated by symbiosis with a specific group of soil bacteria commonly referred to as rhizobia. Symbiotic interactions lead to the development of root nodules, specialized organs where nitrogen fixation occurs. The process of nodule formation involves a complex interplay between the plant and bacteria, coupled with profound genetic reprogramming of the host cells. The establishment of symbiosis and nodule development are initiated by an exchange of molecular signals between two organisms. Both processes are influenced by specific phytohormones and coordinated by multiple genes and miRNAs (for recent reviews see Oldroyd et al. 2011; Bazin et al. 2012). However, little is known about the involvement of DCLs in the nodulation process. To date, only three DCL coding genes, *MtDCL1*, *2* and *3*, have been identified in *M. truncatula* (Capitão et al. 2011). In contrast, two other almost fully sequenced legume genomes (*Glycine max* and *Lotus japonicus*) are known to contain a *DCL4* homolog (Bustos-Sanmamed et al. 2013). In general, our knowledge on DCLs in legumes is limited and far from systematized.

In this study, we report the existence of six putative DCL genes in the *M. truncatula* genome. They encode DCL proteins of all four types (DCL1–4), including three DCL2 isoforms. All six genes are expressed in various plant tissues and significantly upregulated in root nodules. Furthermore, we report the accumulation of an alternative splicing variant of MtDCL1 mRNA. The collected data suggest that this mRNA variant may be recognized and cut by DCL proteins. In addition, it may be involved in a negative feedback regulation mechanism controlling the level of the DCL1 protein as it has been earlier shown for *Arabidopsis*.

## Results and discussion

### Six putative DCL genes are present in the *M. truncatula* genome

**Fig. 1:**
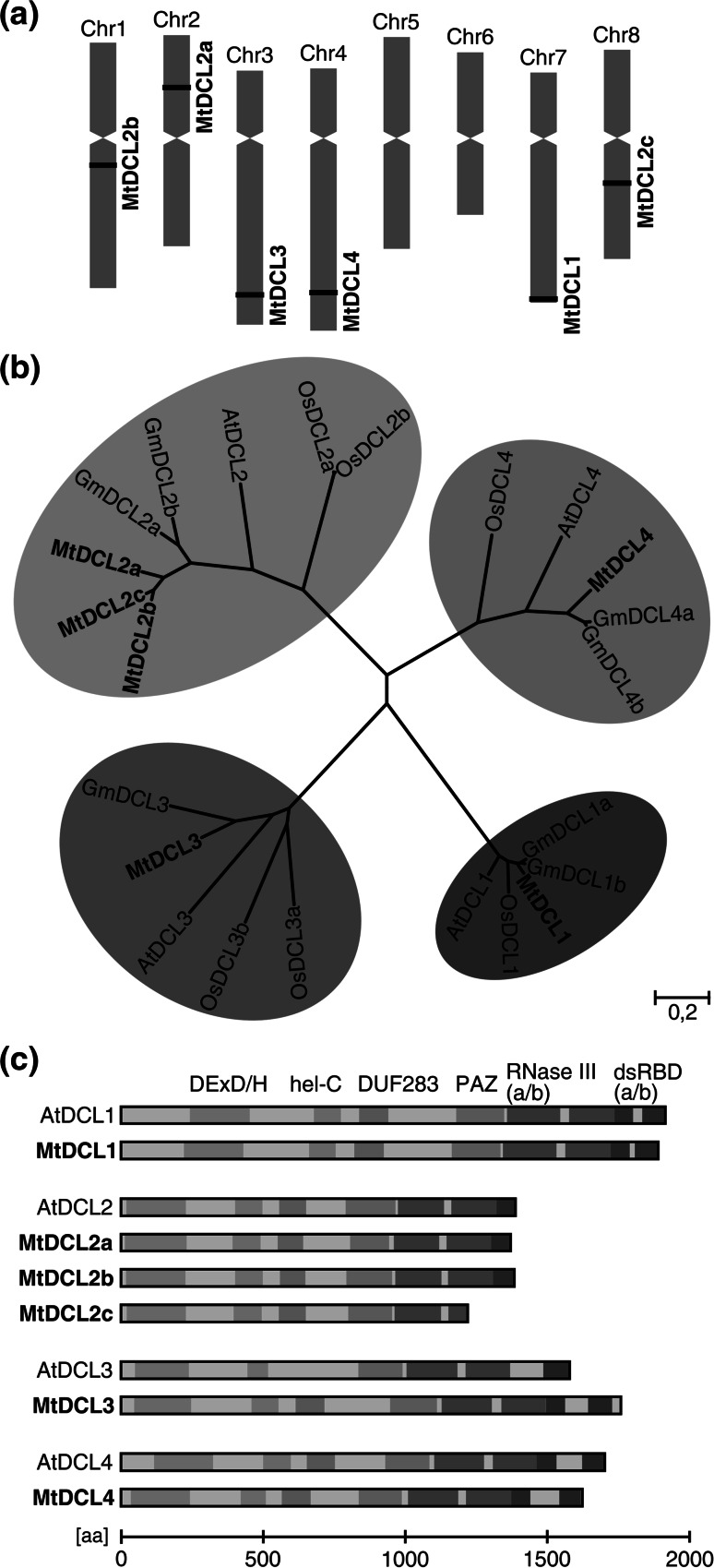
Six putative *DCL* genes are present in the *M. truncatula* genome. **a** Chromosomal localization of *MtDCL* gene loci based on Mt4.0 genome assembly. **b** Maximum-likelihood phylogenetic tree of *DCL* genes in *M. truncatula* (Mt), *G. max* (Gm), *A. thaliana* (At), and *O. sativa* (Os). DCL proteins in plants are classified into four functionally divergent types (DCL1–4) that form distinct clusters within the tree. **c** Domain architecture of the six *M. truncatula* (Mt) DCL proteins compared with their *A. thaliana* (At) counterparts. DCL proteins differ in the number of C-terminal dsRBDs. MtDCL2c is a truncated DCL protein, lacking part of RNase IIIb and dsRBD

The most recent Mt4.0 genome assembly includes extensive gene annotation based on computational, EST and RNA-seq data (Tang et al. 2014). The Mt4.0 assembly was screened with tBLASTn using *Arabidopsis* and soybean DCL protein sequences. As a result, in addition to the previously characterized *MtDCL1*-*3* (Capitão et al. 2011) we were able to identify three additional MtDCL genes. Interestingly, each MtDCL gene is located separately on a different chromosome (Fig. 1a). Predicted coding sequences for all six MtDCL genes were translated and used for phylogenetic tree construction (Fig. 1b). DCL proteins from *Medicago*, *Arabidopsis*, soybean and rice included in the analysis formed four distinct monophyletic groups, each corresponding to one of the four types of AtDCL proteins. Both *DCL1* and *DCL3* occur in a single copy in the *Medicago* genome. Duplication of the latter was identified in rice (Kapoor et al. 2008), sorghum (Liu et al. 2014) and maize (Qian et al. 2011) and is considered as specific to monocots (Margis et al. 2006). Among the three newly identified MtDCL genes one was classified as an *AtDCL4* homolog and designated *MtDCL4*. The presence of the *AtDCL4* homolog in the *M. truncatula* genome has been expected and very preliminarily reported by Bustos-Sanmamed et al. (2013). In *Arabidopsis*, *AtDCL4* was shown to play a critical role in the plant antiviral response (Deleris et al. 2006). AtDCL4 is also involved in the production of phased secondary siRNAs (Xie et al. 2005) which have been shown to be abundant in *Medicago* cells (Zhai et al. 2011). Thus, one can speculate that MtDCL4 plays the same roles in *Medicago*. Coding sequences of two other newly identified MtDCL genes revealed over 95 % identity at nucleotide level and were found to encode DCL2 isoforms. Intriguingly, one gene includes a stop codon within the open reading frame, the use of which would result in production of a protein lacking a portion of the RNase IIIb domain and the whole C-terminal dsRBD. The newly identified *M. truncatula DCL2* homologs were named *MtDCL2b* (coding for full length protein) and *MtDCL2c* (coding for truncated protein). The previously identified *DCL2* homolog was named *MtDCL2a* (Table 1). Duplication of DCL2 genes was observed in other legumes, including *G. max* (Curtin et al. 2012) and *L. japonicus* (Bustos-Sanmamed et al. 2013). However, in contrast to the *G. max* and *L. japonicus DCL2* homologs that occur as uni-directional tandem duplications on one chromosome, the three *M. truncatulaDCL2* copies are separated on three different chromosomes. With the exception of MtDCL2c, the other *Medicago* DCLs exhibit typical domain architecture, including a helicase region with DExD/H-box and Helicase-C, DUF283, PAZ, RNase IIIa/b and dsRBD a/b (or single dsRBD in case of DCL2 homologs, Fig. 1c).

**Table 1:** *DCL* genes and their products in *M. truncatula*

Gene	Locus name	Location coordinates (Mt4.0)	Protein length (aa)	Protein MW (kDa)
*MtDCL1*	Medtr7g118350	Chr7: 49139645–49129819	1880	211.6
*MtDCL2a*	Medtr2g030490	Chr2: 11495894–11486899	1377	156.6
*MtDCL2b*	Medtr1g060740	Chr1: 26454644–26463238	1385	157.5
*MtDCL2c*	Medtr8g069975	Chr8: 29673538–29680711	1219	138.6
*MtDCL3*	Medtr3g105390	Chr3: 48587585–48600236	1730	192.6
*MtDCL4*	Medtr4g116860	Chr4: 48375041–48390001	1625	182.4

### All *M. truncatula* DCL genes are transcriptionally active and significantly induced in root nodules

**Fig. 2:**
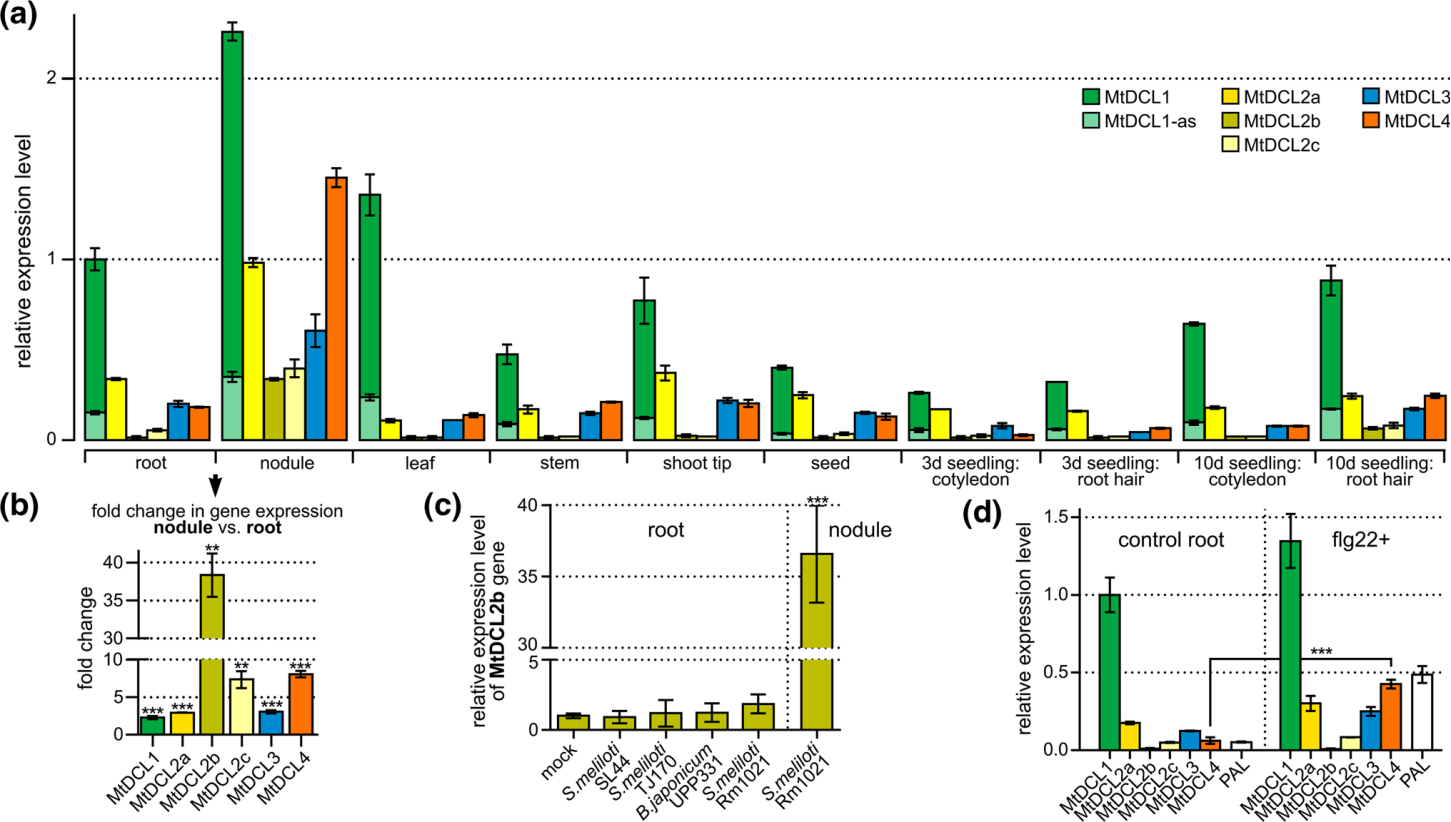
Expression profile of *M. truncatula* *DCL* genes. **a** Expression of *MtDCL* genes was examined with ddPCR in different parts of the plant: cotyledon (with hypocotyl) and root hair of 3- and 10-day-old seedling (seedl), mature leave, stem, shoot tip, root, nodule and seed. Additionally, accumulation of an alternative splicing variant with intron 14 retention (MtDCL1-as) that constitute a fraction of the total *MtDCL1* mRNA was measured. *Bars* represent mean values ± SD of three biological replicates normalized to the expression of the β-actin gene. **b** The mean expression fold change ± SD of the six *M. truncatula* *DCL* genes in nodule compared to root. *Asterisks* denote significant upregulation of a particular gene in nodule in comparison to the level of its expression in root (**P < 0.001, ***P < 0.0001; one-way ANOVA followed by Tukey’s test). **c** The levels of *MtDCL2b* expression in roots and nodules of plants treated with *S. meliloti* Rm1021 and in roots of plants treated with *S. meliloti* mutants SL44 and TJ170 and *B. japonicum* UPP331 strain. *Bars* represent mean values ± SD of three biological replicates normalized to the expression of the β-actin gene. *Asterisks* denote significant upregulation of *MtDCL2b* in nodule in comparison to root (***P < 0.0001; one-way ANOVA followed by Tukey’s test). **d** The levels of *MtDCL* expression in roots treated with flg22 peptide compared to mock-treated control. *Bars* represent mean values ± SD of three biological replicates normalized to the expression of the β-actin gene. Phenylalanineamonialyase (*PAL*) gene was used as a marker of the activation of plant defense responses. *Asterisks* denote significant upregulation of *MtDCL4* under flg22 treatment (***P < 0.0001; paired *t* test)

To assess the expression levels of the six *M. truncatula* DCL genes we used droplet digital PCR (ddPCR), which allows absolute quantification of target transcripts (Hindson et al. 2013). The analyses were performed for different parts of the plant: cotyledon (with hypocotyl) and root hair of 3- and 10-days-old seedlings, respectively, as well as mature leave, stem, shoot tip, root, nodule and seed. We found that all identified DCL genes were expressed in all analyzed organs (Fig. 2a). MtDCL1 mRNA showed the highest accumulation in all analyzed samples. A similar observation was reported for *Arabidopsis* leaves and stems in different developmental stages and roots under various stress conditions (Liu et al. 2009). We also noted a gradual increase of *MtDCL1* expression along plant development. These findings confirm a central role for DCL1 in plant gene regulation. In contrast, the lowest accumulation was always observed for the *MtDCL2b* and *MtDCL2c* transcripts. Interestingly, the mRNA levels of all assayed DCL genes were remarkably increased in nodules compared to other plant organs. We detected over 20-fold increase of *MtDCL2b* expression in nodule in comparison to root (Fig. 2b, c). A greater than sevenfold increase was found for *MtDCL2c* and *MtDCL4*, while expression of the other DCL genes was increased approximately threefold (Fig. 2b). These observations are consistent with previous reports on the involvement of multiple miRNAs in nodule development (Combier et al. 2006; Boualem et al. 2008; Li et al. 2010). Moreover, distinct accumulation patterns of the most typical, i.e. 21 nt, miRNAs, as well as their 22 and 24 nt variants, were observed in *M. truncatula* roots and nodules. Some of the identified 24 nt RNAs were found to be derived from the portion of the precursor that contains miRNA* (Lelandais-Brière et al. 2009). Such diversity in miRNA length suggests that in addition to MtDCL1, other DCL proteins may be involved in miRNA precursor processing during nodule development. Our observation that in *M. truncatula* nodules all DCL genes, in particular *MtDCL2b, MtDCL2c* and *MtDCL4* undergo significant upregulation supports this hypothesis.

The high fold change in MtDCL2b mRNA accumulation between root and nodule suggests a specific role for this gene in nodule development and functional diversification of the MtDCL2 isoforms. In all analyzed samples except nodules *MtDCL2b* transcript was detected at very low levels that could be considered negligible in terms of functional potential. Conversely, in nodules *MtDCL2b* transcript constitute a significant pool of *DCL2*-type transcripts. To better understand the possible role of *MtDCL2b* we analyzed the levels of this gene expression in roots and nodules of *M. truncatula* plants treated with *Sinorhizobium meliloti* (a nitrogen-fixing bacterium that forms a symbiotic relationship with legumes from the genera *Medicago*, *Melilotus* and *Trigonella*) or its symbiotically defective mutants. Our experiments involved symbiotic strain *S. meliloti* Rm1021 and two mutants which do not produce Nod factor (NF): *S. meliloti* SL44 and TJ170 (Fisher et al. 1988). NF is a key signal molecule sent by symbiotic bacterial strains, which initiates a complex signaling pathway in plant roots. Reception of NF triggers transcriptional changes of multiple genes and eventually leads to the nodule formation and symbiosis establishment (Oldroyd et al. 2011; Suzaki et al. 2015). Both SL44 and TJ170 mutants are unable to produce NF and have been shown not to induce the symbiosis-associated changes in the expression of *M. truncatula* genes in roots (Mitra et al. 2004). Our experiment additionally included a set of plants treated with *Bradyrhizobium japonicum* (*B. japonicum* UPP331 strain) which is a known *Lupinus luteus*, but not *M. truncatula* symbiont (Stepkowski et al. 2011). To determine whether *MtDCL2b* is also induced during *Medicago* interactions with pathogenic bacteria we tested the levels of *MtDCL* expression in *M. truncatula* roots treated with flg22 peptide (a fragment of bacterial flagellin from *Pseudomonas aeruginosa*). Flg22 peptide is a known pathogen associated molecular pattern (PAMP) and was shown to elicit specific innate immune response in plants (Felix et al. 1999).

We found that the expression of *MtDCL2b* was significantly induced in nodules only (Fig. 2c) The expression of this gene remained almost unchanged in the roots of plants treated with both symbiotic and non-symbiotic bacterial strains as well as in a mock control. This again suggests a specific role of *MtDCL2b* for nodule development and/or function. *MtDCL2b* expression may be induced together with other genes associated with *S. meliloti* symbiosis or at the later stages of nodule development.

In addition, our experiments showed that flg22 stimulation affected neither the expression of *MtDCL2b* nor the other MtDCL genes except *MtDCL4* (Fig. 2d). This observation confirms that *MtDCL2b* is specifically induced during the nodulation process, and this effect is not associated with merely the presence of either symbiotic or pathogenic bacteria. In contrast, we showed that *MtDCL4* undergoes significant upregulation in nodules of plants treated with *S. meliloti* Rm1021 and in roots of plants treated with flg22 implying that this gene may be involved in both symbiotic interactions and antibacterial defense. A number of *M. truncatula* genes were shown to be involved in both symbiotic and pathogenic interactions, suggesting that the plant employs some common mechanisms in these responses (Mitra et al. 2004). Moreover, modulation of pathogen defense-associated pathways seems to be essential for symbiotic interactions and nodule formation (Bazin et al. 2012). MtDCL4 may be therefore an element of both nodule development and pathogen response pathways. In a similar manner some miRNAs were found to regulate both plant-pathogen interactions and the early stages of symbiosis (Simon et al. 2009).

Significant upregulation of *MtDCL2b* in nodules (compared to *MtDCL2a* and *c*) also indicates functional diversification of MtDCL2 isoforms. Such situation has been previously observed for two rice DCL3 family members that specialize in the production of different classes of 24-nt siRNAs (Song et al. 2012). Additionally, one of the soybean *DCL2* paralogs has been found to be regulated by miR1515, a miRNA involved in nodule formation (Li et al. 2010). In *Medicago*, miR1507 has been shown to target the previously identified DCL2 mRNA (MtDCL2a mRNA) and to trigger phased secondary siRNAs production but it is not known whether it is specific towards all MtDCL2 mRNAs or an individual species (Zhai et al. 2011). Among the three *MtDCL2* copies known today MtDCL2a mRNA still appears to be the best target for miR1507 (in terms of miRNA-mRNA duplex stability; Supporting Information Fig. S1). However, the function of miR1507-triggered secondary siRNAs remains unknown. It is possible that due to high similarity between the three *MtDCL2* paralogs, MtDCL2a mRNA-derived secondary siRNAs regulate the whole gene family, as it is the case for many other phased secondary siRNAs found in plants (Zheng et al. 2015).

### Two MtDCL mRNA variants encode truncated proteins

**Fig. 3:**
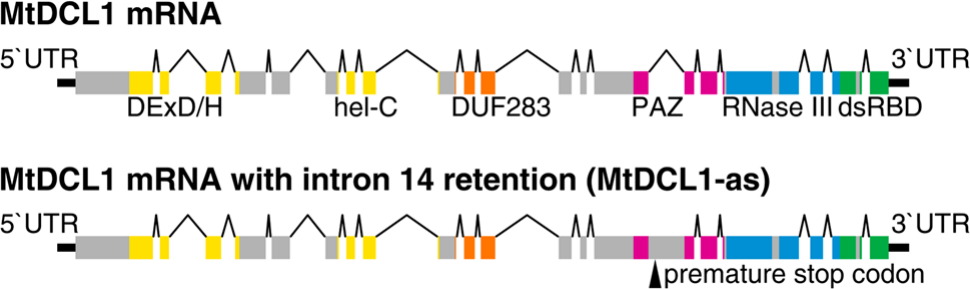
Splicing variants of *MtDCL1* transcripts. Intron 14 retention results in the introduction of a premature stop codon within the MtDCL1 open reading frame

During routine MtDCL1 cDNA cloning we identified a splicing variant of the MtDCL1 mRNA with intron 14 retention. We designated this variant as MtDCL1-as mRNA (Fig. 3). Using ddPCR we determined the levels of MtDCL1-as mRNA accumulation in different parts of the plant, as we did with other MtDCL transcripts (Fig. 2a). MtDCL1-as mRNA constituted a significant fraction of the total MtDCL1 mRNA in all analyzed RNA samples: 9 % in seed and between 15 and 22 % in the remaining parts of the plant (Table S3). Alternative splicing associated with DCL1 intron 14 was also observed in *Arabidopsis* (Xie et al. 2003). Intron 14 in the AtDCL1 transcript (homologous to MtDCL1 intron 14) forms an extended hairpin structure that serves as a precursor of Ath miR838 (Rajagopalan et al. 2006). AtDCL1-mediated cleavage of the Ath miR838 precursor leads to the accumulation of two non-productive fragments of AtDCL1 mRNA. This process prevents proper splicing of a fraction of the AtDCL1 mRNA and functions as a negative feedback regulation mechanism controlling the level of *AtDCL1* expression (Rajagopalan et al. 2006).

We found that MtDCL1 intron 14 may also fold into a similar hairpin structure containing a sequence homologous to Ath miR838 (Fig. S2). Indications of DCL1-mediated MtDCL1-as mRNA cleavage within the intron 14 region would be the accumulation of both non-productive MtDCL1-as mRNA fragments and intron 14-derived small RNAs, as it was observed in *A. thaliana*. Unfortunately, our attempts to identify any MtDCL1-as mRNA fragments with the 5′ end located within intron 14 were unsuccessful (a standard 5′ RACE method was used). However, the analysis of the short RNA libraries that have recently been generated by Formey et al. (2014) showed that three 21 nt-long small RNAs found in *M. truncatula* perfectly matched the stem-loop sequence of intron 14 (Fig. S2). Interestingly, none of these small RNAs was the Ath miR838 homolog. This finding suggests that MtDCL1 intron 14 serves as a substrate for DCL1, as it is the case in *A. thaliana*, but the sequences of the generated short RNAs are not conserved between the two species. The fact that we were able to clone and sequence MtDCL1-as mRNA proves that at least some fraction of the alternatively spliced transcripts remains intact in the cell and encodes a truncated protein. In *A. thaliana* it was proposed that DCL1 competes with the splicing machinery for the DCL1 precursor transcript (Rajagopalan et al. 2006). Therefore, a possibility exists that the same mechanism operates in *M. truncatula*. The variable ratio of MtDCL1-as mRNA to total MtDCL1 mRNA in different plant organs implies that alternative splicing of DCL1 pre-mRNA itself is involved in regulation of the levels of DCL1 protein. For instance, MtDCL1-as mRNAs may compete with the fully spliced transcripts during translation process. The fact that *Medicago* and *Arabidopsis* share a similar DCL1 splicing variant may suggest its evolutionary conservation. It is possible that alternative splicing of DCL1 mRNA, together with conservation of miR162 function, provides a complex regulatory mechanism that controls DCL1 enzyme abundance in various plant species.

The unspliced intron 14 of MtDCL1-as mRNA introduces a premature stop codon within the MtDCL1 open reading frame. As a consequence, MtDCL1-as mRNA encodes a truncated protein containing the helicase domains, DUF283 and part of the PAZ domain. Another truncated DCL protein lacking half of the RNase IIIb domain and C-terminal dsRBD is encoded by the *MtDCL2c*. It is tempting to speculate that the truncated DCLs are produced in plant cells and that these proteins retain some biological function. Examples of this phenomenon were found in other species. For example, in *Caenorhabditis elegans* CED-3 caspase-mediated cleavage of the Dicer protein generates a truncated enzyme encompassing part of the RNase IIIa domain followed by the RNase IIIb and C-terminal dsRBD (Nakagawa et al. 2010). It was shown in vivo and in vitro that the truncated Dicer protein loses the capability to process dsRNA but gains DNase activity and plays a role in DNA fragmentation during apoptosis. The predicted *MtDCL2c* product encoded by the *M. truncatula* genome resembles the truncated *C. elegans* Dicer by containing one fully preserved and one trimmed RNase III domain. The *MtDCL2c*-encoded protein lost the C-terminal dsRBD typically present in plant DCL2-type proteins (Mukherjee et al. 2013). However, the C-terminal dsRBD in human Dicer is required for dsRNA cleavage only in the absence of the PAZ domain (Ma et al. 2012). Alternatively, truncated proteins lacking nuclease activity may regulate the full length protein activity by sequestering the DCL cofactors required for miRNA and siRNA production. For instance, MtDCL1-as-encoded protein contains DUF283 (dimerization domain) which has been shown to be involved in specific interactions with DCL protein partners (Qin et al. 2010).

## Conclusions

In conclusion, we report the existence of six putative *DCL* genes in the *M. truncatula* genome. They are ubiquitously expressed throughout the plant and significantly induced in root nodules. This suggests an important role of DCL genes in nodule function, both in organ development and modulation of symbiont interaction pathways. We found that the *MtDCL2b* is far more upregulated in the nodule than other MtDCL genes, which may suggest a nodule-specific role for the encoded DCL2-type protein and functional diversification of DCL2 isoforms in *M. truncatula*. In contrast, we show that *MtDCL4* undergoes significant upregulation in nodules and in plant roots under flg22 treatment, suggesting that this gene may be involved in both symbiotic interactions and PAMP-triggered immune responses. We also detected the formation of two transcripts (MtDCL2c and MtDCL1-as mRNAs) that encode truncated DCL proteins and accumulate in various plant organs. Alternative splicing of MtDCL1 pre-mRNA may be involved in tissue-specific regulation of DCL1 enzyme abundance. The question of whether truncated DCLs are produced and the biological role these proteins may play awaits further elucidation.

## Methods

### Identification and characterization of putative DCL genes in the *M. truncatula* genome

The tBLASTn algorithm was used to search the Mt4.0 *M. truncatula* genome assembly (Tang et al. 2014) for DCL genes. *A. thaliana* and *G. max* DCL protein sequences downloaded from NCBI GenBank were used as queries (Supporting Information Table S1). All hits considered as candidate genes were searched against the NCBI Conserved Domain Database (CDD; Marchler-Bauer et al. 2011) to confirm the presence of typical Dicer domains. Predicted spliced transcript sequences based on computational, EST and RNA-seq data were downloaded from the Mt4.0 database. The longest open reading frames were translated and the molecular weights were calculated using Expasy Proteomics server tools. The domain architectures were examined using the CDD and EMBL SMART (Letunic et al. 2012) databases. A multiple sequence alignment and phylogenetic analysis were performed with MEGA 6 (Tamura et al. 2013). DCL protein sequences from *M. truncatula*, *A. thaliana*, *G. max* and *O. sativa* species were aligned using ClustalW. A phylogenetic tree was constructed using the maximum likelihood method with 1000 bootstraps. Evolutionary distances were computed using the Jones-Taylor-Thornton (JTT) matrix-based model.

### Plant material, growth and treatment conditions

*Medicago truncatula* seeds (cv. Gaertn, ecotype Jemalong J5) were sterilized and germinated on plates with damp filter paper. The plate-grown 3- and 10-day-old seedlings were used as a source of cotyledon (with hypocotyl) and young root samples, respectively. To obtain mature plants, 7-day-old seedlings were transferred from plates to vermiculite for 2 weeks, and then each plant was individually transferred to a 0.5 L pot with perlite supplemented with sand. Plants were grown in a greenhouse (thermoperiod of 25/18 °C, photoperiod of 16:8 h (day:night), relative humidity of 40 % and a photosynthetic photon fluorescence rate (PPFR) of 500 μmol m^−2^ s^−1^; plants were watered once per 48 h and supplemented with N-P-K fertilizer (6:3:6) once per week). Pot grown plants were a source of leaf, root, stem, shoot tip and seed samples. To obtain mature nodules and roots treated with different S*. meliloti* and *B. japonicum* strains plants were grown on buffered nodulation medium (BNM) plates (Ehrhardt et al. 1992). The 7-day-old seedlings were inoculated with 200 µL per root of a washed suspension of bacterial culture (OD_600_ = 0.5 in 10 mM MgSO_4_) and grown for 3 weeks. For the flg22 experiment 7-day-old seedlings were transferred to 0.5 MS agar plates and grown for 10 days. Roots were incubated in 1 μM solution of flg22 (GenScript) or water (mock control; 100 μl per root) for 1 h, washed with water and collected 24 h after treatment. Samples from 3 biological replicates were obtained and in each case the collected material was immediately frozen in liquid nitrogen and stored at −80 °C.

### Droplet digital PCR (ddPCR) analysis

RNA was isolated from ground plant material using the RNeasy Plant Mini Kit (Qiagen) with on-column DNase (Qiagen) treatment and analyzed on an Agilent 2100 Bioanalyzer. RNA samples with RIN ≥8.0 were used for cDNA synthesis by reverse transcription with the Omniscript Reverse Transcription Kit (Qiagen), oligo-dT primers and 500 ng of RNA according to manufacturer’s instructions. Reaction mixtures for ddPCR containing cDNA samples diluted 1:50–1:500, 200 nM primers (listed in Table S2) and QX200 ddPCR EvaGreen Supermix (Bio-Rad) were emulsified with the QX200 Droplet Generator (Bio-Rad) and subjected to thermal cycling as follows: enzyme activation at 95 °C for 5 min, 40 cycles of 95 °C for 30 s, 57 °C for 30 s and 72 °C for 45 s, then signal stabilization at 4 °C for 5 min and 90 °C for 5 min. Positive and negative fluorescent droplets in each ddPCR reaction were detected with the QX200 Droplet Reader (Bio-Rad). Data were analyzed using the QuantaSoft software (Bio-Rad), and the relative mRNA expression in each sample was normalized to actin mRNA content (as described by Limpens et al. 2005), which has proved beneficial in ddPCR analyses with a low number of repeats or during the detection of low-fold changes in gene expression (Zmienko et al. 2015). Statistical analyses were performed using Prism 6 software (GraphPad). One-way ANOVA followed by Tukey’s post hoc test was used to assess significant differences in multiple comparisons.

### cDNA cloning

MtDCL1 cDNA was amplified from the stem tip and total cDNA samples were prepared as for ddPCR. Primer design was based on the predicted spliced transcript sequence derived from genomic locus Medtr7g118350 (Table S2). The Fast Start High Fidelity PCR System (Roche) was used to amplify the fully spliced *MtDCL1* transcript and the transcript with intron 14 retention. PCR products were cloned using the TOPO XL PCR Cloning Kit (Life Technologies) and sequenced.

### Computational analysis

For miRNA-target analyses including minimum free energy calculations RNAhybrid server was used (Rehmsmeier et al. 2004). The processed short RNA-seq reads by Formey et al. (2014) were downloaded from the NCBI GEO database (GSE49226) and mapped to the Mt4.0 genome assembly using CLC Genomics Workbench 8.5 software (CLC bio).

#### **Author contribution statement**

AT, AU and MF conceived the design of the study. AT and JP performed the experiments. AT performed all the bioinformatics work. AT and JP sampled plant material. AT performed ddPCR experiments and did the statistical analyses. JP performed the MtDCL1 cDNA cloning. AU and MF supervised the research. AT and AU wrote the draft of the manuscript and MF is responsible for its final form. AK-K and NK assisted in editing the article. All authors read and approved the final manuscript.

## Electronic supplementary material

Below is the link to the electronic supplementary material.

Supplementary material 1 (DOC 627 kb)
