# Supplementary material for: Six Medicago truncatula Dicer-like protein genes are expressed in plant cells and upregulated in nodules
Source: Plant Cell Rep. 2016 Jan 29;35:1043–52. doi: 10.1007/s00299-016-1936-8 (PMC4833791; doi:10.1007/s00299-016-1936-8)
Supplement: Supplementary file 1 — Supplementary material 1 (DOC 627 kb) [file 299_2016_1936_MOESM1_ESM.doc]

**Supplementary Material:**

**
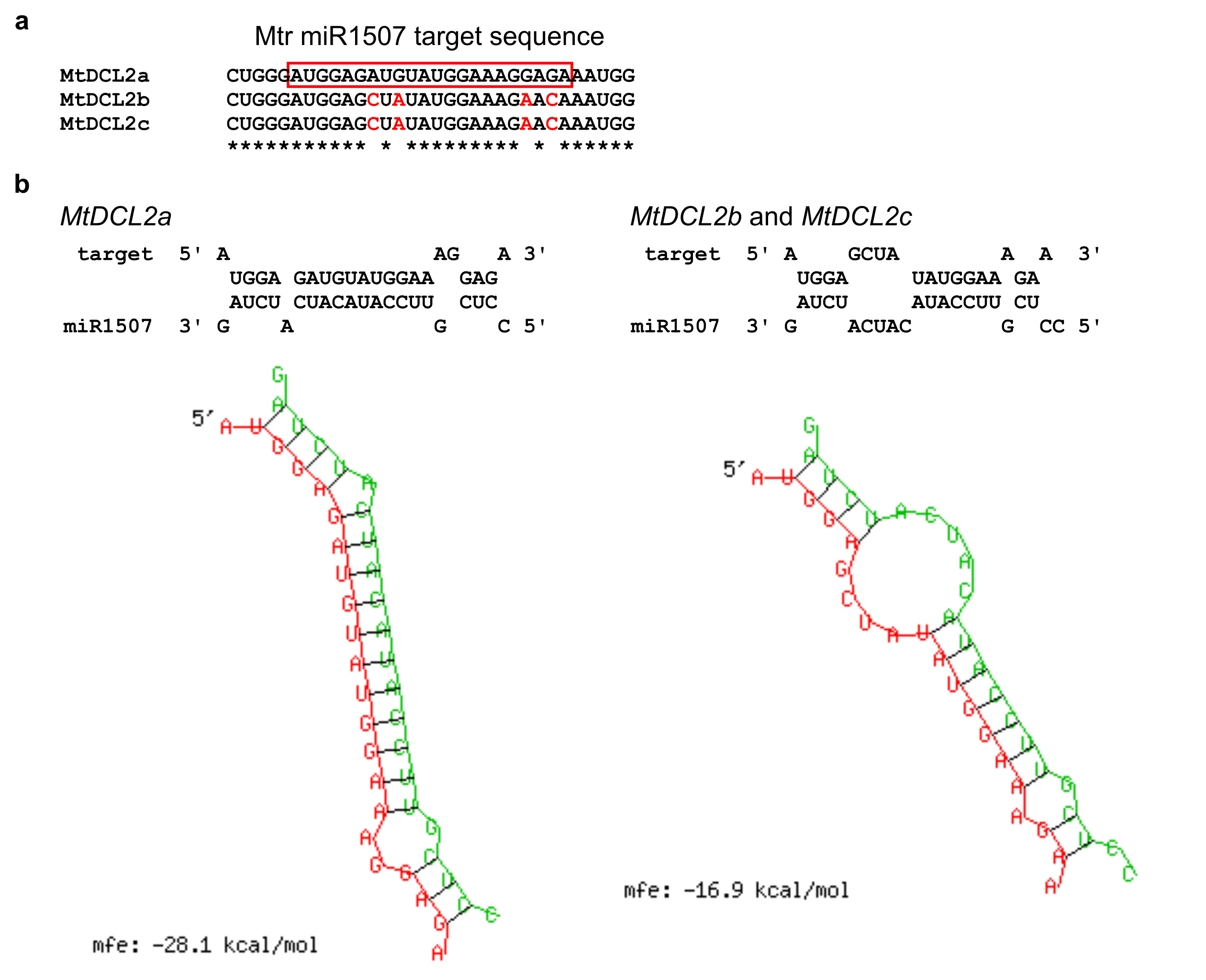
**

**Fig. S1.** A. Alignemnt of MtDCL2a transcript sequence targeted by miR1507 (red box) and corresponding sequences from two other MtDCL2 genes. *MtDCLb* and *c* sequences are identical in this region. B. Comparison of Mtr miR1507-target duplex structures for the corresponding sequences found in the three MtDCL2 genes. MtDCL2a mRNA is a better target for Mtr miR1507 than the other two genes. Mfe – minimum free energy.

**
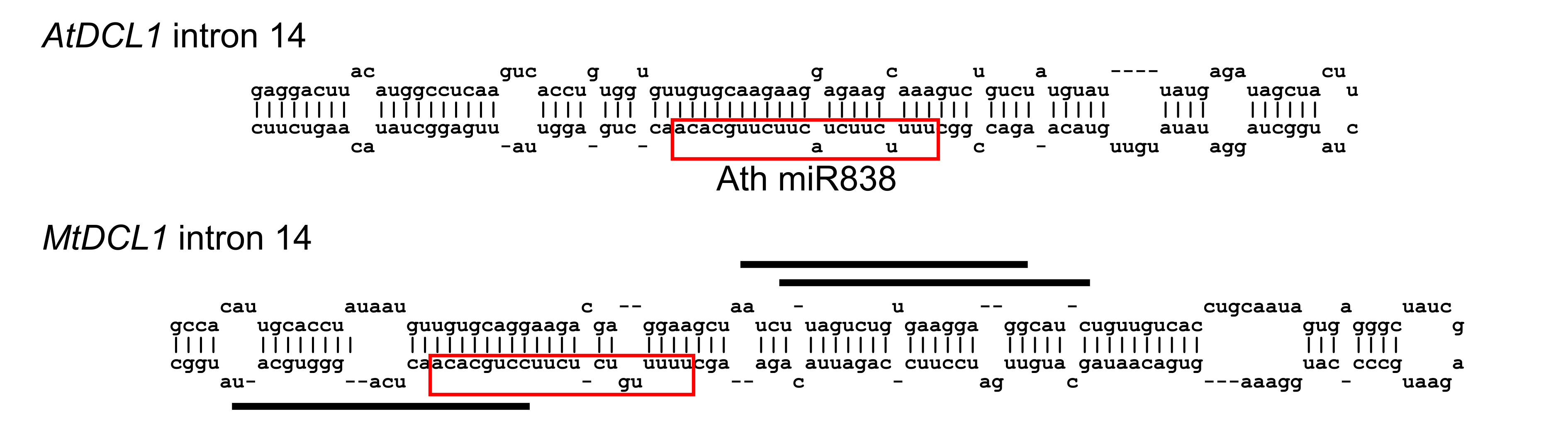
**

**Fig. S2.** Stem-loop structures formed by intron 14 sequences present in aberrantly spliced AtDCL1 and MtDCL1 transcripts. *A. thaliana* miR838 and homologous sequence from *M. truncatula* are marked in red. Black lines represent three short RNAs (21nt long) identified in *M. truncatula* short RNAs library from Formey et al 2014, which perfectly match the stem-loop sequence.

**Table S1** Sequences of *A. thaliana* (At), *G. max* (Gm) and *O. sativa* (Os) DCL proteins used in the study.

| **Protein** | **Sequence name** |
| --- | --- |
| **AtDCL1** | At1g01040.2 |
| **AtDCL2** | At3g03300.1 |
| **AtDCL3** | At3g43920.2 |
| AtDCL4 | At5g20320.1 |
| GmDCL1a | Glyma19g45060.1 |
| GmDCL1b | Glyma03g42290.1 |
| GmDCL2a | Glyma09g02930.1 |
| GmDCL2b | Glyma09g02920.1 |
| GmDCL3 | Glyma04g06060.1 |
| GmDCL4a | Glyma13g22450.1 |
| GmDCL4b | Glyma17g11235.1 |
| OsDCL1 | LOC_Os03g02970.1 |
| **OsDCL2a** | LOC_Os03g38740.1 |
| **OsDCL2b** | LOC_Os09g14610.1 |
| **OsDCL3a** | LOC_Os01g68120.1 |
| **OsDCL3b** | LOC_Os10g34430.1 |
| **OsDCL4** | LOC_Os04g43050.1 |

**Table S2** Oligonucleotides used in the study.

| **Sequence (5`-3`)** | **Usage** |
| --- | --- |
| GCTGCTTTGAAAGAGAAGGAA | ddPCR (MtDCL1) |
| CTCACACACCGGTAGAATGG | ddPCR (MtDCL1) |
| GGCTACTGGTTCTCTTTTACTGC | ddPCR (MtDCL1-as) |
| CATCTTCATGTCCTTCACTGTGT | ddPCR (MtDCL1-as) |
| GATGCTACCAGCGACTTGAA | ddPCR (MtDCL2a) |
| TATTCACCGATGCAGACCTC | ddPCR (MtDCL2a) |
| GATGCTTGTCCAGACAATCTAATC | ddPCR (MtDCL2b) |
| AAGTCGCTGATAGCATCTTGG | ddPCR (MtDCL2b) |
| ATCAATTCTTGTCCAGACAATCTTC | ddPCR (MtDCL2c) |
| TCAAGTCTCTGATAGCATCTAGGC | ddPCR (MtDCL2c) |
| CAGTAGCAATTAAAAAGCCCAAAT | ddPCR (MtDCL3) |
| TGATGAAACAACTGGAATAGAACC | ddPCR (MtDCL3) |
| AGTAGTTGTGAAGCAACTTTAGCAG | ddPCR (MtDCL4) |
| ATTTGGAATTGGAGCTTCTGA | ddPCR (MtDCL4) |
| TTCTCTCAGTACTTTCCAGC | ddPCR (actin) |
| AAGCATCACAATCACTCC | ddPCR (actin) |
| GAAGCAACCTTTTGTTTGAGC | ddPCR (PAL) |
| ACGATACTCCTCCACCATACG | ddPCR (PAL) |
| TAGAATAGGCGTTGATACACAGCAATAGG | MtDCL1 cloning |
| ACAACCACTGCTTGCTTCTGATTGG | MtDCL1 cloning |

**Table. S3** The percentage of MtDCL1-as in the total MtDCL1 mRNA fraction in various plant tissues.

| **Plant tissue** | **Percentage of the MtDCL1-as  in the total MtDCL1 mRNA fraction** |
| --- | --- |
| seed | 9 % |
| 3-days old seedling hypocotyl | 22 % |
| 3-days old seedling root hair | 19 % |
| 10-days old seedling hypocotyl | 15 % |
| 10-days old seedling root hair | 19 % |
| Leaf | 17 % |
| Stem | 19 % |
| Shoot tip | 16 % |
| Root | 15 % |
| Nodule | 15 % |
